# A Generalized Measure of Cumulative Residual Entropy

**DOI:** 10.3390/e24040444

**Published:** 2022-03-23

**Authors:** Sudheesh Kumar Kattumannil, E. P. Sreedevi, Narayanaswamy Balakrishnan

**Affiliations:** 1 Applied Statistics Unit, Indian Statistical Institute, Chennai 600029, India; 2 Department of Statistics, SNGS College, Pattambi 679306, India; sreedeviep@gmail.com; 3 Department of Mathematics and Statistics, McMaster University, Hamilton, ON L8S 4K1, Canada; bala@mcmaster.ca

**Keywords:** cumulative entropy, cumulative residual entropy, extropy, Tsallis entropy, weighted cumulative residual entropy

## Abstract

In this work, we introduce a generalized measure of cumulative residual entropy and study its properties. We show that several existing measures of entropy, such as cumulative residual entropy, weighted cumulative residual entropy and cumulative residual Tsallis entropy, are all special cases of this generalized cumulative residual entropy. We also propose a measure of generalized cumulative entropy, which includes cumulative entropy, weighted cumulative entropy and cumulative Tsallis entropy as special cases. We discuss a generating function approach, using which we derive different entropy measures. We provide residual and cumulative versions of Sharma–Taneja–Mittal entropy and obtain them as special cases this generalized measure of entropy. Finally, using the newly introduced entropy measures, we establish some relationships between entropy and extropy measures.

## 1. Introduction

The uncertainty associated with a random variable can be evaluated using information measures. In many practical situations in lifetime data analysis, experimental physics, econometrics and demography, measuring the uncertainty associated with a random variable is very important. The seminal work on information theory started with the concept of Shannon entropy or differential entropy introduced by Shannon (1948) [1]. For an absolutely continuous non-negative random variable *X*, the differential entropy is given by

$$H\left(X\right)=-E\left(\log f\left(X\right)\right)=-\int_{0}^{\infty }f\left(x\right)\log f\left(x\right)dx,$$

where f(x) is the probability density function of *X* and “log” stands for the natural logarithm, with 0log0 taken as 0.

Several measures of entropy have been introduced in the literature since then, each one being suitable for some specific situations. The widely used measures of entropy are cumulative residual entropy (CRE) [2], cumulative entropy (CE) [3] and the corresponding weighed measures by Mirali et al. (2016) [4] and Mirali and Baratpour (2017) [5]. A unified formulation of entropy has been put forward by Balakrishnan et al. (2022) [6] recently. For a non-negative random variable *X* with distribution function F(x), the cumulative residual entropy, which measures the uncertainty in the future of a lifetime of a system, is defined as

(1) $$E\left(X\right)=-\int_{0}^{\infty }\bar{F}\left(x\right)\log\bar{F}\left(x\right)dx,$$

where $\bar{F}\left(x\right)=1-F\left(x\right)$ is the survival function of *X*. Asadi and Zohrevand (2007) [7] gave a representation of (1) based on the mean residual life function as

E(X)=E(r(X)),

where r(t) is the mean residual life function of *X* at time *t* given by

$$r\left(t\right)=E\left(X-t|X>t\right)=\frac{\int_{t}^{\infty }\bar{F}\left(u\right)du}{\bar{F}\left(t\right)}.$$

Di Crescenzo and Longobardi (2009) [3] introduced cumulative entropy for estimating the uncertainty in the past lifetime of a system as

(2) $$\mathrm{CE}\left(X\right)=-\int_{0}^{\infty }F\left(x\right)\log F\left(x\right)dx.$$

The weighted versions of E(X) and CE(X) have been studied in the literature as well. Weighted cumulative residual entropy, introduced by Mirali et al. (2016) [4], is defined as

(3) $$E^{w}\left(X\right)=-\int_{0}^{\infty }x\bar{F}\left(x\right)\log\bar{F}\left(x\right)dx.$$

Mirali and Baratpour (2017) [5] introduced weighted cumulative entropy as

(4) $${\mathrm{CE}}^{w}\left(X\right)=-\int_{0}^{\infty }xF\left(x\right)\log F\left(x\right)dx.$$

A detailed discussion on weighted entropies has been made by Suhov and Sekeh (2015) [8]. Some additional recent developments in this area were due to [9,10,11,12].

Recently, Kharazmi and Balakrishnan (2020) [13] proposed Jensen cumulative residual entropy, which is an extension of (1). Kharazmi and Balakrishnan (2020) [14] then studied cumulative residual and relative cumulative residual Fisher information measures and their properties. More general cumulative residual-information-generating and relative cumulative residual-information-generating measures have been introduced and studied by Kharazmi and Balakrishnan (2021) [15]. Fractional generalized cumulative entropy and its dynamic version have been proposed by Di Crescenzo et al. (2021) [16].

Several extensions of Shannon entropy are available in the literature, obtained by introducing some additional parameters so that these measures become sensitive to different characteristics and shapes of probability distributions. One important generalization of Shannon entropy is due to Tsallis (1988) [17], known as generalized Tsallis entropy of order α. For a continuous random variable *X*, the generalized Tsallis entropy of order α is defined as [17]

$$T_{\alpha }\left(X\right)=\frac{1}{\alpha -1}\left(1-\int_{0}^{\infty }f^{\alpha }\left(x\right)dx\right),\;\alpha \neq 1.$$

Many extensions or modifications have also been provided for $T_{\alpha }\left(X\right)$. Sati and Gupta (2015) [18] proposed a cumulative Tsallis residual entropy of order α, and Rajesh and Sunoj (2019) [19] modified it and defined cumulative residual Tsallis entropy of order α as

(5) $$CRT_{\alpha }\left(X\right)=\frac{1}{\alpha -1}\int_{0}^{\infty }\left(\bar{F}\left(x\right)-{\bar{F}}^{\alpha }\left(x\right)\right)dx,\;\alpha >0,\;\alpha \neq 1.$$

For a non-negative continuous random variable *X*, Chakraborty and Pradhan (2021) [20] defined weighted cumulative residual Tsallis entropy (WCRTE) of order α as

(6) $$WCRT_{\alpha }\left(X\right)=\frac{1}{\alpha -1}\int_{0}^{\infty }x\left(\bar{F}\left(x\right)-{\bar{F}}^{\alpha }\left(x\right)\right)dx,\;\alpha >0,\;\alpha \neq 1.$$

They also introduced dynamic weighted cumulative residual Tsallis entropy of order α.

Calì et al. (2017) [9] introduced cumulative Tsallis past entropy as

(7) $$CT_{\alpha }\left(X\right)=\frac{1}{\alpha -1}\int_{0}^{\infty }\left(F\left(x\right)-F^{\alpha }\left(x\right)\right)dx,\;\alpha >0,\alpha \neq 1.$$

Calì et al. (2021) [21] subsequently introduced a family of mean past weighted entropies of order α, using the concept of mean inactivity time. Chakraborty and Pradhan (2021) [20] defined weighted cumulative Tsallis entropy (WCTE) of order α as

(8) $$WCT_{\alpha }\left(X\right)=\frac{1}{\alpha -1}\int_{0}^{\infty }x\left(F\left(x\right)-F^{\alpha }\left(x\right)\right)dx,\;\alpha >0,\alpha \neq 1.$$

They also studied dynamic weighted cumulative Tsallis entropy of order α. As is evident from the description above, several entropy measures are available in the literature. Recently, Balakrishnan et al. (2022) [22] have provided a unified formulation of entropy and demonstrated its applications.

In the present work, we define a generalized cumulative residual entropy and study its properties in Section 2. We show that cumulative residual entropy, weighted cumulative residual entropy, cumulative residual Tsallis entropy and weighted cumulative residual Tsallis entropy are all special cases of the proposed measure. We also propose a new generalized cumulative entropy measure and discuss some of its properties. We use the generating function approach to obtain some new entropy measures. In Section 3, we provide cumulative (residual) versions of Sharma–Taneja–Mittal entropy and obtain them as special cases of the generalized measures of entropy introduced in Section 2. In Section 4, we establish some relationships between entropy and extropy measures. Finally, we make some concluding remarks in Section 5.

## 2. Generalized Cumulative Entropy

In this section, we introduce generalized cumulative (residual) entropy measures. We then show that several entropy measures are special cases of the proposed entropies. Some generalizations of CRE and CE have been discussed in the literature and we now review these briefly. Drissi et al. (2008) [23] generalized the definition of CRE, given by Rao et al. (2004) [2], to the case of distributions with general support. They also showed that this generalized CRE can be used as an alternative to differential entropy. Kayal (2016) [24] introduced a generalization of CE proposed by Di Crescenzo and Longobardi (2009) [3] and their dynamic versions. Their definition is related to lower records and the reversed relevation transform. Psarrakos and Navarro (2013) [25] proposed a generalized cumulative residual entropy (GCRE), related to record values from a sequence of independent and identical random variables and with the relevation transform. Some properties and applications of the GCRE in actuarial risk measures have been discussed by Psarrakos and Toomaj (2017) [26]. Under some assumptions, Navarro and Psarrakos (2017) [27] proved that the GCRE function of a fixed order *n* uniquely determines the distribution function. Consequently, some characterizations of particular probability models have been obtained from this general result. Di Crescenzo and Toomaj (2017) [28] obtained some further results associated with generalized cumulative entropy, such as stochastic orders, bounds and characterization results. Some characterizations for the dynamic generalized cumulative entropy have also been derived. Recently, Di Crescenzo et al. (2021) [16] proposed the fractional generalized cumulative entropy and its dynamic version.

We introduce here generalized CRE and CE, which encompass most of the existing variations of these measures, as demonstrated below.

### 2.1. Generalized Cumulative Residual Entropy

Let *X* be a non-negative random variable with absolutely continuous distribution function F(x) and $\bar{F}\left(x\right)$ as the survival function. We assume that the mean μ=E(X) is finite.

**Definition** **1.** *Let X be a non-negative random variable with absolutely continuous distribution function F. Further, let ϕ(.) be a function of X and w(.) be a weight function. Then, the generalized cumulative residual entropy of X is defined as*

(9) $$\mathrm{GE}\left(X\right)=\int w\left(u\right)E\left[\phi (X)-\phi (u)|X>u\right]dF\left(u\right),$$

*where w and ϕ can be chosen arbitrarily under the existence of the above integral such that GE(X) becomes concave.*

Entropy is defined as a measure of uncertainty associated with a model. Strict concavity implies that entropy will increase under averaging. The general definition of entropy we have given contains two arbitrary functions ϕ and *w*, and though it is difficult to state general conditions under which concavity of the general measure will hold, one can chose these functions so that GE(X) becomes concave.

However, with different choices of weight function w(.) and ϕ(.), we can introduce several measures of entropy. First, we show that the new measure reduces to the cumulative residual entropy of Rao et al. (2004) [2] and the weighted cumulative residual entropy of Mirali et al. (2016) [4] for some specific choices of ϕ(.) and w(.).

Using integration by parts, from (1), we obtain (see [29])

$$\begin{array}{ccc} E(X) & = & \int_{0}^{\infty }x\left[-\log\bar{F}\left(x\right)\right]dF\left(x\right)-E\left(X\right). \end{array}$$

Let us denote the hazard rate of *X* by

$$\lambda \left(x\right)=\frac{f(x)}{\bar{F}\left(x\right)},$$

where *f* is the density function of *X*. Then, the cumulative hazard function Λ(x) can be expressed as

$$\Lambda \left(x\right)=\int_{0}^{x}\lambda \left(u\right)du=-\log\bar{F}\left(x\right).$$

Now, with w(u)=1 and ϕ(x)=x, the expression in (9) becomes

$$\begin{array}{ccc} \mathrm{GE}(X) & = & \int_{0}^{\infty }E\left(X-u|X>u\right)dF\left(u\right)\\ & = & \int_{0}^{\infty }E\left(X|X>u\right)f\left(u\right)du-\int_{0}^{\infty }udF\left(u\right)\\ & = & \int_{0}^{\infty }\frac{f(u)}{\bar{F}\left(u\right)}\int_{u}^{\infty }xdF\left(x\right)du-E\left(X\right)\\ & = & \int_{0}^{\infty }x\int_{0}^{x}\frac{f(u)}{\bar{F}\left(u\right)}dudF\left(x\right)-E\left(X\right)\\ & = & \int_{0}^{\infty }x\int_{0}^{x}\lambda \left(u\right)dudF\left(x\right)-E\left(X\right)\\ & = & \int_{0}^{\infty }x\left[-\log\bar{F}\left(x\right)\right]dF\left(x\right)-E\left(X\right). \end{array}$$

Thus, GE(X) reduces in this case to the cumulative residual entropy of Rao et al. (2004) [2].

The weighted cumulative residual entropy defined in (3) can be written as [29]

$$\begin{array}{ccc} E^{w}\left(X\right) & = & \frac{1}{2}\int_{0}^{\infty }x^{2}\left[-\log\bar{F}\left(x\right)\right]dF\left(x\right)-\frac{1}{2}E\left(X^{2}\right). \end{array}$$

If we choose w(u)=1/2 and $\phi \left(x\right)=x^{2}$ and proceed as above, we can show that (9) becomes

$$\begin{array}{ccc} \mathrm{GE}(X) & = & \frac{1}{2}\int_{0}^{\infty }E\left(X^{2}-u^{2}|X>u\right)dF\left(u\right)\\ & = & \frac{1}{2}\int_{0}^{\infty }x^{2}\left[-\log\bar{F}\left(x\right)\right]dF\left(x\right)-\frac{1}{2}E\left(X^{2}\right). \end{array}$$

Thus, GE(X) reduces in this case to the weighted cumulative residual entropy, $E^{w}\left(X\right)$.

Next, we show that the cumulative residual Tsallis entropy of order α is a special case of GE(X). An alternative representation of $CRT_{\alpha }\left(X\right)$ is [19]

$$CRT_{\alpha }\left(X\right)=E\left(r\left(X\right){\bar{F}}^{\alpha -1}\left(X\right)\right),$$

where r(X)=E(X−x|X>x). If we now choose $w\left(u\right)={\bar{F}}^{\alpha -1}\left(u\right)$ and ϕ(x)=x, (9) becomes

$$\mathrm{GE}\left(X\right)=\int_{0}^{\infty }E\left(X-u|X>u\right){\bar{F}}^{\alpha -1}\left(u\right)dF\left(u\right)=E\left(r\left(X\right){\bar{F}}^{\alpha -1}\left(X\right)\right).$$

Chakraborty and Pradhan (2021) [20] expressed a weighted version of cumulative residual Tsallis entropy of order α in (6) as

(10) $$WCRT_{\alpha }\left(X\right)=\int_{0}^{\infty }\frac{1}{\bar{F}\left(t\right)}\int_{t}^{\infty }x\bar{F}\left(x\right)dx{\bar{F}}^{\alpha -1}\left(t\right)dF\left(t\right).$$

Upon noting that the integral

$$\begin{array}{ccc} \frac{1}{\bar{F}\left(t\right)}\int_{t}^{\infty }x\bar{F}\left(x\right)dx & = & \frac{1}{\bar{F}\left(t\right)}\int_{t}^{\infty }x\int_{x}^{\infty }dF\left(y\right)dx\\ & = & \frac{1}{\bar{F}\left(t\right)}\int_{t}^{\infty }\int_{t}^{y}xdxdF\left(y\right)\\ & = & \frac{1}{\bar{F}\left(t\right)}\int_{t}^{\infty }\frac{1}{2}\left(y^{2}-t^{2}\right)dF\left(y\right)\\ & = & \frac{1}{2}E\left(X^{2}-t^{2}|X>t\right), \end{array}$$

(10) becomes

$$WCRT_{\alpha }\left(X\right)=\frac{1}{2}\int_{0}^{\infty }E\left(X^{2}-t^{2}|X>t\right){\bar{F}}^{\alpha -1}\left(t\right)dF\left(t\right).$$

Now, for the choices of $w\left(u\right)={\bar{F}}^{\alpha -1}\left(u\right)$ and $\phi \left(x\right)=\frac{1}{2}x^{2}$, from (9), we obtain the above expression. Thus, $WCRT_{\alpha }\left(X\right)$ is a special case of GE(X) as well. The special cases of GE(X) discussed here are all listed in Table 1.

Next, we derive expressions for GE(X) for some specific distributions.

Consider the exponential distribution with mean λ. Then, it is well-known that mean residual life is equal to mean. Thus, when w(u)=1, we have

$$\mathrm{GE}\left(X\right)=\int_{0}^{\infty }w\left(u\right)\lambda dF\left(u\right)=\lambda .$$

In general, GE(X) is a constant for any weight function. For the standard exponential, taking $\phi \left(x\right)=x^{2}$, we have

$$\begin{array}{ccc} \mathrm{GE}(X) & = & \int_{0}^{\infty }w\left(u\right)E\left(X^{2}-u^{2}|X>u\right)e^{-u}du\\ & = & \int_{0}^{\infty }w\left(u\right)\left(u+1\right)e^{-u}du. \end{array}$$

Thus, GE(X)=2 when w(u)=1 and GE(X)=1.5 when $w\left(u\right)=\bar{F}\left(u\right)$.

Next, let us consider the standard uniform distribution with pdf f(x)=1, 0<x<1. Then,

$$\begin{array}{ccc} \mathrm{GE}(X) & = & \int_{0}^{1}w\left(u\right)\frac{1}{1-u}\int_{u}^{1}\left(1-x\right)dxdu\\ & = & \int_{0}^{1}w\left(u\right)\frac{1}{2(1-u)}{\left(u-1\right)}^{2}du\\ & = & \int_{0}^{1}w\left(u\right)\left(1-u\right)du. \end{array}$$

We thus obtain the residual entropy as $\frac{1}{4}$. Additionally, when w(u)=1 and $\phi \left(x\right)=x^{2}/2$, we get

$$\begin{array}{ccc} \mathrm{GE}(X) & = & \frac{1}{6}\int_{0}^{1}\left(1-u\right)\left(2u+1\right)du=\frac{5}{36}, \end{array}$$

as given in Example 5 of Balakrishnan et al. (2022) [29].

### 2.2. Generalized Cumulative Entropy

In this sub-section, we introduce a generalized cumulative entropy and discuss some of its properties.

**Definition** **2.** *Let X be a non-negative random variable with absolutely continuous distribution function F. Further, let ϕ(.) be a function of X and w(.) be a weight function. Then, the generalized cumulative entropy is defined as*

(11) $$\mathrm{GCE}\left(X\right)=\int_{0}^{\infty }w\left(u\right)E\left[\phi (u)-\phi (X)|X\leq u\right]dF\left(u\right),$$

*where w and ϕ can be chosen arbitrarily under the existence of the above integral such that GCE(X) becomes concave.*

For the choices of w(u)=1 and ϕ(x)=x, (11) reduces to CE(X) [3]. Similarly, GCE(X) in (11) reduces to the weighted cumulative entropy of Mirali and Baratpour (2017) [5] for the choices of w(u)=1/2 and $\phi \left(x\right)=x^{2}$.

The reversed hazard rate function of *X*, denoted by h(.), is defined as

$$h\left(x\right)=\frac{f(x)}{F(x)},$$

which yields the cumulative reversed hazard rate function as

$$H\left(x\right)=\int_{x}^{\infty }\frac{f(u)}{F(u)}du=-\log F\left(x\right).$$

The cumulative entropy in (2) can be expressed as (see [29])

$$\begin{array}{ccc} \mathrm{CE}(X) & = & \int_{0}^{\infty }x\left[-\log F\left(x\right)\right]dF\left(x\right)+E\left(X\right). \end{array}$$

Thus, by using the cumulative reversed hazard rate function, we can express

$$\begin{array}{ccc} \mathrm{CE}(X) & = & -\int_{0}^{\infty }x\left[\log F\left(x\right)\right]dF\left(x\right)+E\left(X\right)\\ & = & E\left(X\right)-\int_{0}^{\infty }x\int_{x}^{\infty }\frac{f(u)}{F(u)}dudF\left(x\right)\\ & = & E\left(X\right)-\int_{0}^{\infty }\frac{1}{F(u)}\int_{0}^{u}xdF\left(x\right)dF\left(u\right)\\ & = & \int_{0}^{\infty }E\left(u-X|X\leq u\right)dF\left(u\right), \end{array}$$

which is the special case of the generalized cumulative entropy in (11) for the choices of w(u)=1 and ϕ(x)=x. Proceeding similarly, we can show that GCE(X) reduces to the weighted cumulative entropy [5] for the choices of w(u)=1/2 and $\phi \left(x\right)=x^{2}$.

Next, we show that the cumulative Tsallis entropy of order α is a special case of GCE(X) in (11). The mean inactivity time function of a random variable *X*, at time *x*, is defined as

$$m\left(x\right)=E\left(x-X|X\leq x\right)=\frac{1}{F(x)}\int_{0}^{x}ydF\left(y\right).$$

Using m(x), $CT_{\alpha }\left(X\right)$ can be expressed as [9]

$$CT_{\alpha }\left(X\right)=E\left(m\left(X\right)F^{\alpha -1}\left(X\right)\right).$$

Now, for the choices of $w\left(u\right)=F^{\alpha -1}\left(u\right)$ and ϕ(x)=x, (11) yields

$$\mathrm{GCE}\left(X\right)=\int_{0}^{\infty }F^{\alpha -1}\left(u\right)E\left[u-X|X\leq u\right]dF\left(u\right)=E\left(m\left(X\right)F^{\alpha -1}\left(X\right)\right).$$

An alternative expression for the weighted cumulative Tsallis entropy of order α is given by [20]

(12) $$WCT_{\alpha }\left(X\right)=\int_{0}^{\infty }\frac{1}{F(t)}\int_{t}^{\infty }xF\left(x\right)dxF^{\alpha -1}\left(t\right)dF\left(t\right).$$

As in Section 2, simple algebraic manipulations yield

$$WCT_{\alpha }\left(X\right)=\frac{1}{2}\int_{0}^{\infty }E\left(t^{2}-X^{2}|X\leq t\right)F^{\alpha -1}\left(t\right)dF\left(t\right).$$

Again, for the choices of $w\left(u\right)=F^{\alpha -1}\left(u\right)$ and $\phi \left(x\right)=\frac{1}{2}x^{2}$, (11) yields

$$\mathrm{GCE}\left(X\right)=\frac{1}{2}\int_{0}^{\infty }E\left[u^{2}-X^{2}|X\leq u\right]F^{\alpha -1}\left(u\right)dF\left(u\right).$$

Thus, $WCT_{\alpha }\left(X\right)$ is a special case of GCE(X). In Table 2, we list the cumulative entropies derived from GCE(X).

Next, we derive expressions for GCE(X) for some specific distributions. Consider the standard exponential distribution with mean λ=1. Then, for the choices of w(u)=1 and ϕ(x)=x, we have

$$\begin{array}{ccc} \mathrm{GCE}(X) & = & \int_{0}^{\infty }\frac{1}{1-e^{-u}}\int_{0}^{u}\left(u-x\right)e^{-x}dxdF\left(u\right)\\ & = & \int_{0}^{\infty }\frac{1}{1-e^{-u}}(u-\left(1-e^{-u}\right)e^{-u}du\\ & = & \int_{0}^{\infty }\frac{1}{1-e^{-u}}ue^{-u}du-1=\frac{\pi^{2}}{6}-1. \end{array}$$

Next, let us consider the standard uniform distribution with pdf f(x)=1, 0<x<1. Then, for the choices of w(u)=1 and ϕ(x)=x, we obtain

$$\begin{array}{ccc} \mathrm{GCE}(X) & = & \int_{0}^{1}\frac{1}{u}\int_{0}^{u}\left(u-x\right)dxdu\\ & = & \frac{1}{2}\int_{0}^{1}udu=1/4. \end{array}$$

These two examples have been presented earlier by Balakrishnan et al. (2022) [29].

### 2.3. Generating Function

We now introduce generating function related to generalized entropy measures discussed in the preceding sections.

**Definition** **3.** *Let X be a non-negative random variable with absolutely continuous distribution function F. Further, let ϕ(.) be a function of X and w(.) be a weight function. We then define a generating function for the generalized cumulative residual entropy measure as*

(13) $$Gf_{re}\left(t\right)=\int_{0}^{\infty }w\left(u\right)E\left(\left[\exp\left(t\phi (X)\right)-\exp\left(t\phi (u)\right)\right]|X>u\right)dF\left(u\right).$$

Being a function of *t*, we can interpret $Gf_{re}\left(t\right)$ as a generating function for the general entropy measure introduced in Section 2. If we differentiate the expression in (13) with respect to *t* once, we obtain

$$Gf_{re}^{{}^{\prime }}\left(t\right)=\int_{0}^{\infty }w\left(u\right)E\left(\left[\phi \left(X\right)\exp\left(t\phi (X)\right)-\phi \left(u\right)\exp\left(t\phi (u)\right)\right]|X>u\right)dF\left(u\right).$$

Now, by setting t=0 in the above expression, we obtain the generalized entropy measure in (9). We, therefore, refer to it as generalized cumulative residual entropy of order 1. Higher-order derivatives with respect to *t* would similarly give rise to generalized cumulative residual entropies of orders 2, 3 and so on. For example, the generalized cumulative residual entropy of order two is given by

(14) $$Gf_{re}\left(2\right)=\int_{0}^{\infty }w\left(u\right)E\left(\phi^{2}\left(X\right)-\phi^{2}\left(u\right)|X>u\right)dF\left(u\right).$$

For the choice of w(u)=1/2 and ϕ(x)=x, $Gf_{re}\left(2\right)$ reduces to the weighted cumulative residual entropy of Mirali et al. (2016) [4].

In a similar manner, we define the generating function for the generalized cumulative entropy as follows.

**Definition** **4.** *Let X be a non-negative random variable with absolutely continuous distribution function F. Further, let ϕ(.) be a function of X and w(.) be a weight function. We then define the generating function for the generalized cumulative entropy as*

(15) $$Gf_{ce}\left(t\right)=\int_{0}^{\infty }w\left(u\right)E\left(\left[\exp\left(t\phi \left(u\right)-\exp\left(t\phi (X)\right)\right)\right]|X\leq u\right)dF\left(u\right).$$

Once again, differentiating (15) with respect to *t* once and setting t=0, we obtain the generalized cumulative entropy (of order 1) measure in (11). From (15), we can similarly obtain the generalized cumulative entropy of order 2 to be

(16) $$Gf_{ce}\left(2\right)=\int_{0}^{\infty }w\left(u\right)E\left(\phi^{2}\left(u\right)-\phi^{2}\left(X\right)|X\leq u\right)dF\left(u\right).$$

The weighted cumulative entropy of Mirali and Baratpour (2017) [5] can be obtained from (16) for the choices of w(u)=1/2 and ϕ(x)=x. Naturally, higher-order derivatives with respect to *t* would give rise to generalized cumulative entropies of orders 3, 4 and so on.

## 3. Sharma–Taneja–Mittal Entropy

In this section, we introduce the cumulative (residual) versions of Sharma–Taneja–Mittal (STM) entropy. We then show that these are indeed special cases of the generalized residual and the past entropy introduced in Section 2.

Sharma and Taneja (1975) [30] and Mittal (1975) [31] independently introduced an entropy of the form

(17) $$S_{\alpha ,\beta }=\frac{1}{\beta -\alpha }\int_{0}^{\infty }\left(f^{\alpha }\left(x\right)-f^{\beta }\left(x\right)\right)dx,\;\;\alpha ,\beta >0,\;\left(\alpha ,\beta \right)\neq \left(1,1\right).$$

For different choices of α and β, from (17), we obtain some entropy measures discussed in the literature. In particular, for α=1−κ and β=1+κ, we obtain Kaniadakis entropy [32], as a special case of Sharma-Mittal entropy. For more details, see [33] along with Table 1 of Ilic et al. (2021) [34].

### 3.1. Sharma–Taneja–Mittal Cumulative Residual Entropy

In this sub-section, we introduce the cumulative residual version of the STM entropy.

**Definition** **5.** *Let X be a non-negative random variable with absolutely continuous survival function $\bar{F}$. Then, the cumulative residual STM entropy is defined as*

(18) $$SR_{\alpha ,\beta }=\frac{1}{\beta -\alpha }\int_{0}^{\infty }\left({\bar{F}}^{\alpha }\left(x\right)-{\bar{F}}^{\beta }\left(x\right)\right)dx,\;\;\alpha ,\beta >0,\;\left(\alpha ,\beta \right)\neq \left(1,1\right).$$

We also introduce the weighted cumulative residual STM entropy as follows.

**Definition** **6.** *Let X be a non-negative random variable with absolutely continuous survival function $\bar{F}$. Then, the cumulative weighted residual STM entropy is defined as*

(19) $$SRW_{\alpha ,\beta }=\frac{1}{\beta -\alpha }\int_{0}^{\infty }x\left({\bar{F}}^{\alpha }\left(x\right)-{\bar{F}}^{\beta }\left(x\right)\right)dx,\;\;\alpha ,\beta >0,\;\left(\alpha ,\beta \right)\neq \left(1,1\right).$$

Next, we show that $SR_{\alpha ,\beta }$ and $SRW_{\alpha ,\beta }$ are indeed special cases of GE(X). Consider

(20) $$\begin{array}{ccc} SR_{\alpha ,\beta } & = & \frac{1}{\beta -\alpha }\int_{0}^{\infty }\left({\bar{F}}^{\alpha }\left(x\right)-{\bar{F}}^{\beta }\left(x\right)\right)dx\\ & = & \frac{1}{\beta -\alpha }\int_{0}^{\infty }\left(\bar{F}\left(x\right)-{\bar{F}}^{\beta }\left(x\right)-\left(\bar{F}\left(x\right)-{\bar{F}}^{\alpha }\left(x\right)\right)\right)dx. \end{array}$$

Now, consider

(21) $$\begin{array}{ccc} \int_{0}^{\infty }\left(\bar{F}\left(x\right)-{\bar{F}}^{\beta }\left(x\right)\right)dx & = & \int_{0}^{\infty }\bar{F}\left(x\right)\left(1-{\bar{F}}^{\beta -1}\left(x\right)\right)dx\\ & = & \int_{0}^{\infty }\bar{F}\left(x\right)\int_{0}^{x}\left(\beta -1\right){\bar{F}}^{\beta -2}\left(t\right)dF\left(t\right)dx\\ & = & \left(\beta -1\right)\int_{0}^{\infty }{\bar{F}}^{\beta -2}\left(t\right)\int_{x}^{\infty }\bar{F}\left(x\right)dxdF\left(t\right)\\ & = & \left(\beta -1\right)\int_{0}^{\infty }{\bar{F}}^{\beta -1}\left(t\right)\frac{1}{\bar{F}\left(t\right)}\int_{t}^{\infty }\bar{F}\left(x\right)dxdF\left(t\right)\\ & = & \left(\beta -1\right)\int_{0}^{\infty }{\bar{F}}^{\beta -1}\left(t\right)r\left(t\right)dF\left(t\right)\\ & = & \left(\beta -1\right)E(r\left(X\right){\bar{F}}^{\beta -1}\left(X\right)). \end{array}$$

Similarly, we obtain

(22) $$\begin{array}{ccc} \int_{0}^{\infty }\left(\bar{F}\left(x\right)-{\bar{F}}^{\alpha }\left(x\right)\right)dx & = & \left(\alpha -1\right)E(r\left(X\right){\bar{F}}^{\alpha -1}\left(X\right)). \end{array}$$

Upon substituting (21) and (22) in (20), we obtain

(23) $$\begin{array}{ccc} SR_{\alpha ,\beta } & = & \frac{1}{\beta -\alpha }E\left(r\left(X\right)\left(\left(\beta -1\right){\bar{F}}^{\beta -1}\left(X\right)-\left(\alpha -1\right){\bar{F}}^{\alpha -1}\left(X\right)\right)\right). \end{array}$$

Now, for the choices of $w\left(u\right)=\frac{1}{\beta -\alpha }\left(\left(\beta -1\right){\bar{F}}^{\beta -1}\left(u\right)-\left(\alpha -1\right){\bar{F}}^{\alpha -1}\left(u\right)\right)$ and ϕ(x)=x, from (9), we obtain $SR_{\alpha ,\beta }$.

By following the same steps as performed for (23), the cumulative weighted residual STM entropy can be expressed as

(24) $$SRW_{\alpha ,\beta }=\frac{1}{\beta -\alpha }\int_{0}^{\infty }E\left(X^{2}-x^{2}|X>x\right)\left(\left(\beta -1\right){\bar{F}}^{\beta -1}\left(x\right)-\left(\alpha -1\right){\bar{F}}^{\alpha -1}\left(x\right)\right)dF\left(x\right).$$

Again, for the choices of $w\left(u\right)=\frac{1}{\beta -\alpha }\left(\left(\beta -1\right){\bar{F}}^{\beta -1}\left(u\right)-\left(\alpha -1\right){\bar{F}}^{\alpha -1}\left(u\right)\right)$ and $\phi \left(x\right)=x^{2}/2$, from (9), we arrive at (24).

### 3.2. Sharma–Taneja–Mittal Cumulative Entropy

In this sub-section, we introduce cumulative and weighted cumulative STM entropies, and then show that they are indeed special cases of the generalized entropy.

**Definition** **7.** *Let X be a non-negative random variable with absolutely continuous distribution function F. Then, the cumulative STM entropy is defined as*

(25) $$SP_{\alpha ,\beta }=\frac{1}{\beta -\alpha }\int_{0}^{\infty }\left(F^{\alpha }\left(x\right)-F^{\beta }\left(x\right)\right)dx,\;\;\alpha ,\beta >0,\;\left(\alpha ,\beta \right)\neq \left(1,1\right).$$

In this case, the weighted cumulative STM entropy is defined as follows.

**Definition** **8.** *Let X be a non-negative random variable with absolutely continuous distribution function F. Then, the cumulative weighted STM entropy is defined as*

(26) $$SPW_{\alpha ,\beta }=\frac{1}{\beta -\alpha }\int_{0}^{\infty }x\left(F^{\alpha }\left(x\right)-F^{\beta }\left(x\right)\right)dx,\;\;\alpha ,\beta >0,\;\left(\alpha ,\beta \right)\neq \left(1,1\right).$$

Of course, in the special cases of α=1−κ and β=1+κ, the above definitions would result in the corresponding generalized versions of Kaniadakis entropy. Following the same steps as those used to obtain alternative expressions for $SP_{\alpha ,\beta }$ and $SPW_{\alpha ,\beta }$, we can express $SP_{\alpha ,\beta }$ and $SPW_{\alpha ,\beta }$, respectively, as

$$\begin{array}{ccc} SP_{\alpha ,\beta } & = & \frac{1}{\beta -\alpha }E\left(m\left(X\right)\left(\left(\beta -1\right){\bar{F}}^{\beta -1}\left(X\right)-\left(1-\alpha \right){\bar{F}}^{\alpha -1}\left(X\right)\right)\right), \end{array}$$

(27) $$SPW_{\alpha ,\beta }=\frac{1}{\beta -\alpha }\int_{0}^{\infty }E\left(x^{2}-X^{2}|X\leq x\right)\left(\left(\beta -1\right){\bar{F}}^{\beta -1}\left(x\right)-\left(1-\alpha \right){\bar{F}}^{\alpha -1}\left(x\right)\right)dF\left(x\right).$$

Now, let $w\left(u\right)=\frac{1}{\beta -\alpha }\left(\left(\beta -1\right){\bar{F}}^{\beta -1}\left(u\right)-\left(\alpha -1\right){\bar{F}}^{\alpha -1}\left(u\right)\right)$. Then, from (11), we obtain $SP_{\alpha ,\beta }$ and $SPW_{\alpha ,\beta }$ in (27) and (27) by taking ϕ(x)=x and $\phi \left(x\right)=x^{2}/2$, respectively.

## 4. Connection between Entropy and Extropy

Apart from entropy, extropy and its properties have also been studied for quantifying the uncertainty associated with a random variable *X*. Using the new entropy measures introduced in the preceding sections, we establish some relationships between entropy and extropy measures in this section.

For a non-negative random variable *X*, extropy is defined as [35]

$$J\left(X\right)=-\frac{1}{2}\int_{0}^{\infty }f^{2}\left(x\right)dx.$$

Now, we briefly discuss some recent developments associated with extropy measures. Jahanshahi et al. [36] defined the cumulative residual extropy as

(28) $$\mathrm{CRJ}\left(X\right)=-\frac{1}{2}\int_{0}^{\infty }{\bar{F}}^{2}\left(x\right)dx,$$

and the cumulative extropy is defined as [37]

(29) $$\mathrm{CJ}\left(X\right)=-\frac{1}{2}\int_{0}^{\infty }\left(1-F^{2}\left(x\right)\right)dx.$$

Sudheesh and Sreedevi (2022) [38] discussed non-parametric estimation of CRJ(X) and CJ(X) for right-censored data.

Recently, Balakrishnan et al. [29], Bansal and Gupta [39] and Sathar and Nair [40,41,42] introduced different weighted versions of extropy. The weighted version of the survival extropy is given by [41]

$$J\left(X,w\right)=-\frac{1}{2}\int_{0}^{\infty }x{\bar{F}}^{2}\left(x\right)dx.$$

These authors also introduced the weighted version of the cumulative extropy as

$$H\left(X,w\right)=-\frac{1}{2}\int_{0}^{\infty }x\left(1-F^{2}\left(x\right)\right)dx,$$

and Sathar and Nair [42] subsequently defined dynamic survival extropy as

$$J_{t}\left(X\right)=-\frac{1}{2{\bar{F}}^{2}\left(t\right)}\int_{t}^{\infty }{\bar{F}}^{2}\left(x\right)dx.$$

For various properties of $J_{t}\left(X\right)$, one may see [36]. Sudheesh and Sreedevi (2022) [38] proposed simple alternative expressions for different extropy measures. Using these expressions, they established relationships between different dynamic and weighted extropy measures, and reliability concepts. In particular, they expressed $J_{t}\left(X\right)$ as

(30) $$J_{t}\left(X\right)=-\frac{1}{2}E\left(\min\left(X_{1},X_{2}\right)-t|\min\left(X_{1},X_{2}\right)>t\right).$$

Thus, $-2J_{t}\left(X\right)$ is the mean residual life function of a series system having two identical components.

Sathar and Nair [41] defined weighted dynamic survival extropy as

$$J_{t}\left(X,w\right)=-\frac{1}{2{\bar{F}}^{2}\left(t\right)}\int_{t}^{\infty }x{\bar{F}}^{2}\left(x\right)dx.$$

Kundu (2021) [43] introduced dynamic cumulative extropy as

$$H_{t}\left(X\right)=-\frac{1}{2F^{2}\left(t\right)}\int_{0}^{t}F^{2}\left(x\right)dx,$$

while Sathar and Nair [41] defined the weighted dynamic cumulative extropy as

$$H_{t}\left(X,w\right)=-\frac{1}{2F^{2}\left(x\right)}\int_{0}^{t}xF^{2}\left(x\right)dx.$$

Sudheesh and Sreedevi (2022) [38] expressed $H_{t}\left(X\right)$ as

(31) $$H_{t}\left(X\right)=-\frac{1}{2}E\left(t-\max\left(X_{1},X_{2}\right)|\max\left(X_{1},X_{2}\right)\leq t\right).$$

Thus, $-2H_{t}\left(X\right)$ is the mean past life function of a parallel system having two identical components, where the mean past life function of a random variable *X* is defined as E(t−X|X≤t).

Next, we establish some connections between different entropy and extropy measures. For the choice of $w\left(u\right)=\bar{F}\left(u\right)$ and ϕ(x)=x, from (9), we obtain

$$\mathrm{GE}\left(X\right)=\int_{0}^{\infty }\bar{F}\left(u\right)F\left(u\right)du=\int_{0}^{\infty }\bar{F}\left(u\right)du-\int_{0}^{\infty }{\bar{F}}^{2}\left(u\right)du.$$

Thus, using (28), we have the relationship

GE(X)=2CRJ(X)+E(X).

Again, for the choices of $w\left(u\right)=\bar{F}\left(u\right)$ and $\phi \left(x\right)=x^{2}$, from (9), we obtain

$$\mathrm{GE}\left(X\right)=\int_{0}^{\infty }2u\bar{F}\left(u\right)F\left(u\right)du=\int_{0}^{\infty }2u\bar{F}\left(u\right)du-\int_{0}^{\infty }2u{\bar{F}}^{2}\left(u\right)du.$$

For a non-negative random variable *X*, we have

$$E\left(X^{2}\right)=\int_{0}^{\infty }x^{2}dF\left(x\right)=\int_{0}^{\infty }2x\bar{F}\left(x\right)dx.$$

Thus, in this case, we obtain the relationship

$$\mathrm{GE}\left(X\right)=4\mathrm{CRJ}\left(X,w\right)+E\left(X^{2}\right).$$

For the choices of w(u)=−F(u) and ϕ(x)=x, from (12), we obtain

(32) $$\mathrm{GCE}\left(X\right)=-\int_{0}^{\infty }\bar{F}\left(u\right)F\left(u\right)du.$$

Using the identity $1-F^{2}\left(x\right)=\bar{F}\left(x\right)+\bar{F}\left(x\right)F\left(x\right)$, from (29) and (32), we have the relationship

GCE(X)=2CJ(X)+E(X).

Additionally, for the choices of w(u)=−F(u) and $\phi \left(x\right)=x^{2}$, from (12), we obtain

$$\mathrm{GCE}\left(X\right)=-\int_{0}^{\infty }2u\bar{F}\left(u\right)F\left(u\right)du.$$

Thus, in this case, we obtain the relationship

$$\mathrm{GCE}\left(X\right)=4\mathrm{CJ}\left(X,w\right)+E\left(X^{2}\right).$$

Let $X_{1}$ and $X_{2}$ be two independent and identical random variables having the same distribution function *F*. Let $Z=\min(X_{1},X_{2})$ be the lifetime of a series system having two identical components. Using (9), we define the generalized residual entropy associated with *Z* as

(33) $$\mathrm{GE}\left(Z\right)=\int 2w\left(u\right)E\left[\phi (Z)-\phi (u)|Z>u\right]\bar{F}\left(u\right)dF\left(u\right),$$

where ϕ(.) is a function of *Z* and w(.) is a weight function. Now, for the choices of $w\left(u\right)=-\frac{1}{4}$ and ϕ(z)=z, from (33), we obtain

$$\mathrm{GE}\left(Z\right)=-\frac{1}{2}\int E\left[\min\left(X_{1},X_{2}\right)-u|\min\left(X_{1},X_{2}\right)>u\right]\bar{F}\left(u\right)dF\left(u\right).$$

Thus, the generalized residual entropy associated with *Z* is the weighted average of the dynamic survival extropy in (30).

Next, let $Z=\max(X_{1},X_{2})$ be the lifetime of a parallel system having two identical components. Then, the generalized cumulative entropy associated with *Z* is defined as

(34) $$\mathrm{GCE}\left(Z\right)=\int_{0}^{\infty }2w\left(u\right)E\left[\phi (u)-\phi (X)|X\leq u\right]F\left(u\right)dF\left(u\right).$$

Again, for the choices of $w\left(u\right)=-\frac{1}{4}$ and ϕ(z)=z, from (34), we obtain

$$\mathrm{GCE}\left(Z\right)=-\frac{1}{2}\int_{0}^{\infty }E\left[u-\max\left(X_{1},X_{2}\right)|\max\left(X_{1},X_{2}\right)\leq u\right]F\left(u\right)dF\left(u\right).$$

Thus, the generalized cumulative entropy associated with *Z* is the weighted average of the dynamic cumulative extropy in (31).

## 5. Concluding Remarks

In this work, we have introduced two general measures of entropy, viz., generalized cumulative residual entropy and generalized cumulative entropy. Several entropy measures known in the literature were all shown to be special cases of these generalized measures. Cumulative residual entropy, weighted cumulative residual entropy, cumulative residual Tsallis entropy and weighted cumulative residual Tsallis entropy are all special cases of the generalized cumulative residual entropy. Cumulative entropy, weighted cumulative entropy, cumulative Tsallis entropy and weighted cumulative Tsallis entropy are all special cases of the generalized cumulative entropy.

We have presented a generating function approach to obtain generalized measures of higher-order. We have shown that the generalized cumulative residual entropy of order two reduces to the weighted cumulative residual entropy of Mirali et al. (2016) [4]. Moreover, the weighted cumulative entropy of Mirali and Baratpour (2017) [5] is a special case of the generalized cumulative entropy of order two. We have also established some relationships between entropy and extropy measures.

In information theory literature, conditional entropy is the amount of information required to describe the outcome of one random variable *Y*, given the value of another random variable *X*. Conditional entropy, as a measure of information, can be defined through any measure, such as the Shannon entropy measure (denoted by H(Y|X)). The conditional entropy defined through Shannon entropy measure, for example, is given by

$$H\left(Y|X\right)=-\int_{0}^{\infty }\int_{0}^{\infty }f\left(x,y\right)\log\frac{f(x,y)}{f(x)}dydx.$$

In this way, we can define the conditional entropy measures even in a generalized form. The generalized versions we have introduced in the present work can thus be extended to conditional entropy notions. However, we plan to carry out a detailed study of this in our future work. It will be of interest to develop some inferential methods for these measures as well. We are currently working in these directions and hope to report these finding in a future paper.

## Figures and Tables

**Table 1:** Special cases of generalized residual entropy.

Entropy Measure	w(u)	ϕ(x)
Cumulative residual entropy	1	*x*
Weighted cumulative residual entropy	$\frac{1}{2}$	$x^{2}$
Cumulative residual Tsallis entropy	${\bar{F}}^{\alpha -1}\left(u\right)$	*x*
Weighted cumulative Tsallis residual entropy	${\bar{F}}^{\alpha -1}\left(u\right)$	$\frac{x^{2}}{2}$

**Table 2:** Special cases of generalized entropy.

Entropy Measure	w(u)	ϕ(x)
Cumulative entropy	1	*x*
Weighted cumulative entropy	$\frac{1}{2}$	$x^{2}$
Cumulative Tsallis entropy	$F^{\alpha -1}\left(u\right)$	*x*
Weighted cumulative Tsallis entropy	$F^{\alpha -1}\left(u\right)$	$\frac{x^{2}}{2}$

## Data Availability

Not applicable.

## Abbreviations

The following abbreviations are used in this manuscript:

CE	Cumulative entropy
CRE	Cumulative residual entropy
STM	Sharma–Taneja–Mittal
WCRTE	Weighted cumulative residual Tsallis entropy
WCTE	weighted cumulative Tsallis entropy

## References

[1] Shannon C.E. (1948). A mathematical theory of communication. Bell Syst. Tech. J..

[2] Rao M., Chen Y., Vemuri B., Wang F. (2004). Cumulative residual entropy: A new measure of information. IEEE Trans. Inf. Theory.

[3] Di Crescenzo A., Longobardi M. (2009). On cumulative entropies. J. Stat. Plan. Inference.

[4] Mirali M., Baratpour S., Fakoor V. (2016). On weighted cumulative residual entropy. Commun. Stat.-Theory Methods.

[5] Mirali M., Baratpour S. (2017). Some results on weighted cumulative entropy. J. Iran. Stat. Soc..

[6] Balakrishnan N., Buono F., Longobardi M. (2022). A unified formulation of entropy and its application. Phys. A Stat. Mech. Its Appl..

[7] Asadi M., Zohrevand Y. (2007). On the dynamic cumulative residual entropy. J. Stat. Plan. Inference.

[8] Suhov Y., Sekeh S.Y. (2015). Weighted cumulative entropies: An extension of CRE and CE. arXiv.

[9] Calì C., Longobardi M., Ahmadi J. (2017). Some properties of cumulative Tsallis entropy. Phys. A Stat. Mech. Its Appl..

[10] Calì C., Longobardi M., Navarro J. (2020). Properties for generalized cumulative past measures of information. Probab. Eng. Inform. Sci..

[11] Tahmasebi S. (2020). Weighted extensions of generalized cumulative residual entropy and their applications. Commun. Stat.-Theory Methods.

[12] Toomaj A., Di Crescenzo A. (2020). Connections between weighted generalized cumulative residual entropy and variance. Mathematics.

[13] Kharazmi O., Balakrishnan N. (2020). Jensen-information generating function and its connections to some well-known information measures. Stat. Probab. Lett..

[14] Kharazmi O., Balakrishnan N. (2020). Cumulative residual and relative cumulative residual Fisher information and their properties. IEEE Trans. Inf. Theory.

[15] Kharazmi O., Balakrishnan N. (2021). Cumulative and relative cumulative residual information generating measures and associated properties. Commun. Stat.-Theory Methods.

[16] Di Crescenzo A., Kayal S., Meoli A. (2021). Fractional generalized cumulative entropy and its dynamic version. Commun. Nonlinear Sci. Numer. Simul..

[17] Tsallis C. (1988). Possible generalization of Boltzmann-Gibbs statistics. J. Stat. Phys..

[18] Sati M.M., Gupta N. (2015). Some characterization results on dynamic cumulative residual Tsallis entropy. J. Probab. Stat..

[19] Rajesh G., Sunoj S.M. (2019). Some properties of cumulative Tsallis entropy of order *α*. Stat. Pap..

[20] Chakraborty S., Pradhan B. (2021). On weighted cumulative Tsallis residual and past entropy measures. Commun. Stat.-Simul. Comput..

[21] Calì C., Longobardi M., Psarrakos G. (2021). A family of weighted distributions based on the mean inactivity time and cumulative past entropies. Ric. Mat..

[22] Balakrishnan N., Buono F., Longobardi M. (2022). On cumulative entropies in terms of moments of order statistics. Methodol. Comput. Appl. Probab..

[23] Drissi N., Chonavel T., Boucher J.M. (2008). Generalized cumulative residual entropy for distributions with unrestricted supports. Res. Lett. Signal Process..

[24] Kayal S. (2016). On generalized cumulative entropies. Probab. Eng. Inform. Sci..

[25] Psarrakos G., Navarro J. (2013). Generalized cumulative residual entropy and record values. Metrika.

[26] Psarrakos G., Toomaj A. (2017). On the generalized cumulative residual entropy with applications in actuarial science. J. Comput. Appl. Math..

[27] Navarro J., Psarrakos G. (2017). Characterizations based on generalized cumulative residual entropy functions. Commun. Stat.-Theory Methods.

[28] Di Crescenzo A., Toomaj A. (2017). Further results on the generalized cumulative entropy. Kybernetika.

[29] Balakrishnan N., Buono F., Longobardi M. (2020). On weighted extropies. Commun. Stat.-Theory Methods.

[30] Sharma B.D., Taneja I.J. (1975). Entropy of type (*α*,*β*) and other generalized measures in information theory. Metrika.

[31] Mittal D.P. (1975). On some functional equations concerning entropy, directed divergence and inaccuracy. Metrika.

[32] Kaniadakis G. (2001). Non-linear kinetics underlying generalized statistics. Phys. A Stat. Mech. Its Appl..

[33] Lopes A.M., Machado J.A.T. (2020). A review of fractional order entropies. Entropy.

[34] Ilić V.M., Korbel J., Gupta S., Scarfone A.M. (2021). An overview of generalized entropic forms (a). EPL (Europhys. Lett.).

[35] Lad F., Sanfilippo G., Agro G. (2015). Extropy: Complementary dual of entropy. Stat. Sci..

[36] Jahanshahi S.M.A., Zarei H., Khammar A.H. (2020). On cumulative residual extropy. Probab. Eng. Inf. Sci..

[37] Tahmasebi S., Toomaj A. (2020). On negative cumulative extropy with applications. Commun. Stat.-Theory Methods.

[38] Sudheesh K.K., Sreedevi E.P. (2022). Non-parametric estimation of cumulative (residual) extropy with censored observations. Stat. Probab. Lett..

[39] Bansal S., Gupta N. (2020). Weighted extropies and past extropy of order statistics and k-record values. Commun. Stat.-Theory Methods.

[40] Sathar E.A., Nair R.D. (2021). On dynamic weighted extropy. J. Comput. Appl. Math..

[41] Sathar E.A., Nair R.D. (2021). A study on weighted dynamic survival and failure extropies. Commun. Stat.-Theory Methods.

[42] Sathar E.A., Nair R.D. (2021). On dynamic survival extropy. Commun. Stat.-Theory Methods.

[43] Kundu C. (2021). On cumulative residual (past) extropy of extreme order statistics. Commun. Stat.-Theory Methods.

